# Development, Implementation and Evaluation of an Acute Care Physical Therapy ‘Float’ Placement during the COVID-19 Pandemic: A Case Report

**DOI:** 10.3390/ijerph20116038

**Published:** 2023-06-02

**Authors:** Jasdeep Dhir, Amy Connell, Magda McCaughan, Diana Hatzoglou, Daana Ajami, Andrea Fursman, Sarah Wojkowski, Michelle E. Kho

**Affiliations:** 1 School of Rehabilitation Science, Physiotherapy, McMaster University, Institute of Applied Health Science, Room 406, 1400 Main St. W, Hamilton, ON L8S 1C7, Canada; 2 Department of Physiotherapy, St. Joseph’s Healthcare Hamilton, 50 Charlton Ave. E, Hamilton, ON L8N 4A6, Canada

**Keywords:** health professions education, clinical education, work integrated learning, physical therapy, curricula

## Abstract

Clinical education is a mandatory component of physical therapy curricula globally. COVID-19 disrupted clinical education, jeopardizing students’ abilities to meet graduation requirements. The objective of this case report is to outline the development, implementation and evaluation of a multiple clinical instructor (CI), multiple unit, acute care float clinical placement for a final year, entry-level physical therapy student and offer implementation recommendations. This placement included an eight-week, multiple CI (one primary, four supporting), multiple (five) unit clinical placement which was developed between St. Joseph’s Healthcare and the McMaster University Masters of Science (Physiotherapy) Program between 10 August and 2 October 2020. Student evaluations and reflections by the student and CIs were collected and analyzed using interpretive description. Analysis from the reflections revealed six themes: (1) CI and student attributes; (2) increased feasibility; (3) varied exposure; (4) central communication and resources; (5) organization; and (6) managing expectations. An acute care clinical experience is required for students in Canadian entry-to-practice physical therapy programs. Due to COVID-19, placement opportunities were limited. The float placement allowed clinicians to offer supervision despite staff re-deployment and increased organizational and work–life pressures during the pandemic. This model provides an approach to extenuating circumstances and may also increase acute care placements during non-pandemic times for physical therapy and other similarly structured healthcare professions.

## 1. Introduction

Clinical education is an essential element of physical therapy entry-level programs, although specific requirements vary across curricula internationally [1,2]. It accounts for one-third of entry-to-practice Canadian entry-level physical therapy curricula [3]. Canadian physical therapy programs must ensure students obtain a minimum of 1025 h in clinical placements across different settings to meet entry-to-practice standards [3,4]. However, acquiring sufficient clinical placements to meet demand has become increasingly difficult [5,6].

Therapist-reported barriers to student supervision include environmental constraints (e.g., time and space), supervision of challenging students, and decreased autonomy [7]. Increased stress, reduced workplace efficiency, student preparation, and student attitude also influences Clinical Instructor (CI) decisions to supervise students [8,9]. Cls typically represent clinicians providing care in the community across various settings. A CI is oriented to clinical supervision and placement expectations by the host university. According to the Canadian National Physiotherapy Entry-To-Practice Curriculum Guidelines, an acute care setting is ‘Physiotherapy care, as part of an Interprofessional team, provided for patients during an acute illness, an acute exacerbation or a surgical intervention which necessitates admission to an acute care facility’ [3]. All students in Canadian entry-level physiotherapy programs must obtain placement experience in an acute care setting. Traditionally, acute care physiotherapy placements use one-to-one models, i.e., student-to-CI, which would expose a student to the assigned CI’s caseload and hospital unit. To increase the number of acute care placements in physical therapy and mitigate identified barriers, innovative models, such as two students to one supervisor, and the addition of a practice tutor, have been explored [10,11,12]. To increase organizational capacity for clinical supervision, other health care disciplines have explored a combination of one-to-one and collective supervision (e.g., multi-modal placements) as an alternative to the traditional one-to-one supervision model [13].

Securing clinical supervision for students in acute care is typically a challenging task. The COVID-19 pandemic amplified these difficulties following restrictions and early pressures placed on hospitals in 2020 [14]. Physical distancing measures, human resource shortages and significant increases in patient admissions added to challenges for CIs to offer placement supervision. Physical therapy programs faced an urgent need to explore feasible alternative models that did not increase CI stress to meet acute care placement demands. A team approach to clinical supervision could increase the number of clinicians willing and available to offer placements [15] while maintaining quality clinical supervision [16]. Although team models are routinely used in clinical supervision for other professions including social work [13], few reports describe the development, implementation and evaluation of these models within physical therapy.

The float position in healthcare is a staff member who moves across hospital units to meet clinical demands. In nursing, the float staff position offers coverage to many hospital units due to surges in patient numbers or staffing shortages [17]. We describe a physical therapy float clinical placement model where supervision by multiple CIs across multiple hospital units is provided to one senior entry-to-practice MSc (PT) student using a team approach.

## 2. Methods

### 2.1. Authors

The research team includes eight physiotherapists with a variety of skills and experience in physiotherapy practice, education, and research, and who serve overlapping roles. Three of the authors are faculty at a Canadian University, and six are clinicians within a Canadian hospital. Of the three faculty, two are associate professors, and one is an assistant professor. The faculty members hold distinct positions: Director of Clinical Education, Assistant Dean (Physiotherapy), and Researcher and Clinician Scientist. Of the six clinicians, one is a recent graduate (student who completed float placement), three are clinicians in acute or chronic care with several years of clinical experience, one is a Physiotherapy Practice Lead (PPL), and one holds dual roles as a clinician and faculty member. The authors acknowledge that their respective roles and affiliations influence the lens in which these data are collected and presented.

### 2.2. Placement Development

In-person clinical placements for most health professional students were suspended across Canada in March 2020 based on Public Health directives in response to the COVID-19 pandemic [18]. Once placements resumed, securing placements presented significant challenges, particularly in acute care, given the climate of uncertainty and added pressures placed on hospital systems. With the rise in COVID-19 hospitalizations, many physical therapists were re-deployed to meet organizational demands. These increased pressures in acute care limited therapists’ capacities to assume full-time student supervision.

To explore all opportunities for MSc (PT) students to meet program requirements, the McMaster University Acting Director of Clinical Education, Physiotherapy (DCE) and the St. Joseph’s Healthcare Physiotherapy Practice Lead (PPL) explored multiple supervision models. St. Joseph’s Healthcare is an academic tertiary hospital and research healthcare organization in Hamilton, Canada. St. Joseph’s Healthcare is a clinical partner site for the McMaster University Master of Science (Physiotherapy) MSc (PT) program and facilitated almost 100 placements since 2015. The PPL proposed to pilot a float acute care placement with the DCE using a shared supervision model by multiple CIs across multiple hospital units. This placement was developed as a fourth and final placement for a MSc (PT) student. Prior to engaging in this placement, the student would have demonstrated success in all previous clinical placements (three) and academic courses. Upon agreement with this conceptual model, the PPL invited physical therapy staff to express interest in participating as CIs for one final-year MSc (PT) student that would engage in the pilot project.

**Figure 1:**
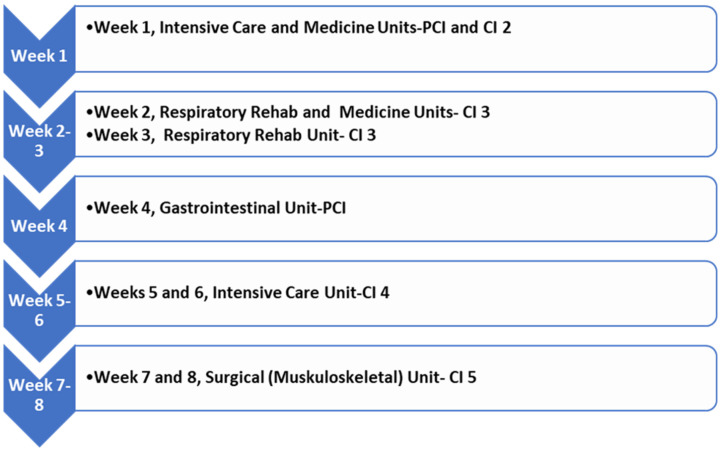
Overview of the eight-week float placement. CI = Clinical Instructor; PCI = Primary Clinical Instructor.

Pre-placement Planning: The PPL developed an outline for the eight-week placement, including unit rotations and CI (Figure 1). Pre-placement, the PPL and the DCE discussed the outline to ensure content and expectations were in alignment with the MSc (PT) program. Five staff physiotherapists, including the PPL, who worked across four different units, agreed to provide supervision as CIs. All CIs received orientation to program expectations. The PPL was the primary CI to facilitate communication and coordinate evaluations. For the remainder of this paper, the PPL/primary CI will be referred to as PCI. The roles of the PCI included developing placement goals, maintaining communications with CIs and the student, completing evaluations and facilitating unit transitions.

Implementation: The full-time placement was eight weeks and included a combination of one- and two-week clinical placement rotations (Figure 1). The student developed overall goals for the placement and unit-specific goals with each CI in a learning contract (LC) (Appendix A). The unit-specific goals were regularly reviewed to ensure they aligned with patient caseload and skills required for the specific placement rotation.

### 2.3. Measures

The Canadian Physiotherapy Assessment of Clinical Performance (ACP) and the LC evaluate students’ placement performance in the McMaster University MSc (PT) program (described below). Evaluations of the ACP and LC occurred at midterm (week four) and at completion (week eight). Unique to this float placement, reflections of the float model by the PCI, CIs and student were also collected.

Clinical Instructor Evaluation of Student Performance

LC: The student, in collaboration with the PCI, carefully considered and developed five overarching SMART (Specific, Measurable, Achievable, Relevant, Time-bound) goals that could be applied to the various experiences included in the float placement (Appendix A). Each of the LC goals are evaluated on a two-point scale. An evaluation of two implies the student has fully met the goal.

ACP: The PCI evaluated the student using the ACP. The ACP is a descriptive measure used across entry-level Canadian Physiotherapy programs by Cls and students to evaluate student behaviors and competencies [19]. To achieve expected entry-level benchmarks on the ACP for a final placement, as outlined by McMaster University, students must demonstrate independence in managing at least 75% of a simple caseload and require minimal supervision for patients with more complex conditions. Students must also conduct comprehensive assessments and intervention planning and demonstrate sound clinical reasoning.

At each transition (i.e., when the student moved to a new unit), an in-person, telephone, or virtual meeting occurred between the PCI and the supervising CI to gather student performance feedback to inform the ACP evaluation. All CIs were oriented to the expected ACP benchmarks set by the Program.

Reflections on the Float Model by PCI, CIs, and Student

Reflective Data Collection: Following each rotation, the DCE contacted all CIs requesting their responses to reflective questions about the placement (Appendix B) in either written or verbal formats. If a CI chose to verbally respond, the DCE arranged a phone call or virtual meeting, transcribed the responses, and invited the CI to review the document for accuracy. The student received the same questions and documented their own reflections and responses at the transition points between units.

Reflective Data Organization and Analysis: The research team used interpretive description to analyze reflections for this case report. Interpretive description is an inductive analytic approach that creates ways of understanding clinical phenomena that result in application implications [20,21,22]. Interpretive description has been used as a methodology in the previous literature to evaluate clinical education in physical therapy [23]. We used interpretive description to guide the process of capturing patterns and themes within subjective perceptions and experiences of the student and CIs within the float placement.

Following the completion of the placement, three authors (JD, AC, MEK) independently reviewed the PCI, CI and student reflections. The three authors independently coded all reflections by highlighting words or segments of text that captured key thoughts or concepts. Each reviewer noted their impressions on initial analysis and identified labels for codes that emerged. Thematic categories were then organized based on coded linkages and relationships. The three authors collaboratively reviewed preliminary coding notes and merged themes until consensus was reached. The frequency of reflections related to a specific theme was also noted. In recognition of the bias that may have influenced data analysis, member checking [24], which included presentation of the initial analysis, was conducted with the entire research team.

Our author group of students (now graduate), CI assessors, and university educators includes power imbalances between individuals. Collection of the student’s placement reflections, co-reviewed by CIs and educators, may have limited the student’s opportunity to provide candid feedback about the strengths and weaknesses of the placement. To help mitigate this concern, we reviewed the student’s reflections after completing all placement rotations and evaluations to ensure that student reflections would have no bearing on CI assessments.

## 3. Results

The student successfully completed and achieved a mark of two on all five goals on the LC. This student met all expected benchmarks for a final placement, set by the MSc (PT) Program, on the ACP final evaluation.

Six themes and associated summaries are identified from the reflections and presented below. The emerging themes are presented from most to least commonly identified: (1) clinical instructor and student attributes; (2) increased feasibility; (3) varied exposure; (4) central communication and resources; (5) organization; and (6) managing expectations.

- Theme 1. Clinical Instructor and Student Attributes

Reflections from both the CIs and student highlighted the importance of being adaptive as a student within this placement model. CIs described the most important student attributes as: open-minded, flexible, adaptive, resilient, motivated, self-directed, have a positive attitude, and demonstrate interest. The student identified that being adaptive as a learner was important and rapid facilitation skills are a necessary CI attribute. Some variation in CI reflections were noted regarding the student level (junior or senior) suggested for the float model.

“...the student and Clinical Instructor have to be: Open minded, able to adapt and adjust, be open to delivering and receiving feedback openly and quickly.”

—PCI

“Student would need to be flexible, adaptable. Would need to be comfortable seeing a wide variety of patients across various settings and use a variety of skill sets.”

—CI 4

“Perhaps consider this model for an earlier placement where the benchmarks and expectations on the evaluations are not as high.”

—CI 5

“Students need to prepare for each unit as they would for the start of a new placement and need to quickly adapt when moving across units. CIs need to be able to facilitate students’ learning and feedback quickly due to a short timeframe.”

—Student

- Theme 2. Increased Feasibility

CIs recognized the increased feasibility in offering a placement using team supervision. Factors identified in increased feasibility included perceived team support and less time required for supervision per CI. The student shared the value of experiencing a broad role and the model increasing supervision support provided, potentially making this a more feasible model for first time CIs.

“This model gave me the opportunity to support a student placement, which would have been impossible in the traditional model.”

—PCI

“The model makes it more feasible to offer a placement.”

—CI 4

For this placement, it was “much more feasible to be able to commit to a shorter supervision period”.

—CI 5

“In the future, I would offer supervision to a student in this model—especially being a first time CI it would be great to have a team of experienced CIs to share and assist the teaching.”

—Student

- Theme 3. Varied Exposure

Varied exposure was a theme identified from both the CIs and student outlining the value of having a placement model that provided experiences across multiple hospital units with the opportunity for varied skill acquisition. The student expressed that increased exposure assisted with confidence, and development of a broad knowledge base across units.

An expected opportunity was the... “Enhanced exposure to other hospital areas/experiences, enhanced exposure to different CIs and skill sets within PT scope (and) identification of common skills that are applied to all areas to broaden depth/scope of understanding of what it’s like to work in hospital”.

—CI 3

“Student could be exposed to many settings/patient populations in one placement period.”

—CI 4

“The key takeaway for me is that I’m really thankful to have the opportunity to practice all my acute care skills with supervision—being across so many units I got to learn and practice chest physio, suctioning, ventilators, oxygen titration, early mobilization, exercises and discharge planning. I think having the opportunity to practice everything you learn is fundamental to being a well-rounded clinician.”

—Student

“It’s great exposure to many units as mentioned above. Also, if one unit is not a student’s strength, it’s nice the student has the opportunity to practice in another unit to be more comfortable and demonstrate their strengths and skill.”

—Student

- Theme 4. Central Communication and Resources

The student and CIs highlighted the critical importance of centralized communication, oversight of evaluations, and coordination of transitions between rotations by the PCI. Suggested communication timepoints with the PCI or within the team included pre-placement, transition periods, and at the end of each placement rotation. A ‘hand off’ communication to assist with transition between CIs was noted as a suggestion for future placements. The student also reflected that receiving additional pre-placement resources, such as specific review materials and a unit specific goal setting process, would allow for shorter onboarding.

“I would consider a brief weekly meeting amongst all instructors working with the student and identify ways to streamline paperwork.”

—CI 2

“Having 1 point contact for the placement organization and for the evaluation was key to the success of this model.”

—CI 4

“Vital to have a point contact for the placement. Would not have been able to evaluate just based on 2 weeks, so it was important that there was one person that had oversight over the entire 8 week placement that did the evaluation.”

—CI 5

“having a specific outline of what the student needs to review before the rotation to shorten onboarding of each unit [would be beneficial]…. [and] perhaps some sort of handoff between CIs—assist with transition between units and continue on the goals from the previous unit; the CI will also have more support between each other with this communication process.”

—Student

- Theme 5. Organization

Reflections from CIs and student also identified the importance of placement organization including considerations for the rotation settings, and time allocated within each rotation. The student and CIs highlighted some challenges with this model, including less time for onboarding the student within each rotation, limited time for the student to develop independent skills, and fewer opportunities for the student to learn patients’ typical recovery patterns across various settings and patient presentation. Two weeks was identified as the minimum duration within a placement rotation, with the student noting the potential benefit to a longer rotation time of two and a half to three weeks for learning skills and meeting expected evaluation benchmarks.

“I would, in the future, try to match the areas a bit better so that the 2 weeks were not such extreme changes in caseload and focus.”

—CI 1

In the future placement organization should ‘consider a graduated approach to working with patients, from less medically complex to more medically complex’.

—CI 2

“Suggest aligning the settings within the placement blocks to they are somewhat similar. This may help with carryover of skills.”

—CI 5

“2 weeks is a good minimum time on a unit; 2.5 or 3 weeks would be great (0.5/1 week for intro, 1 week to practice, 1 week for mastery/independence).”

—Student

- Theme 6. Managing Expectations

Managing expectations also emerged as a theme from reflections. Given the student had less time within each rotation than a typical placement, the student had more opportunities to develop a wide breadth of skills, however fewer opportunities to develop depth in skills. To proactively address this concern, we established explicit and aligned learning goals during the planning phases of the placement. CIs outlined that managing both student and CI expectations was integral to placement success and the student highlighted the importance of managing personal expectations related to performance.

“The CIs need to identify realistic learning goals and patients for the student.”

—CI 2

A potential barrier expected with this model was the “Reduced time not allowing for full development of student skill set and independence”.

—CI 3

The clinical instructor “needs to be able to manage their expectations and goals and have these set early on”.

—CI 5

“This was my first acute, inpatient placement, so I was nervous about not being quick enough to keep up with the expectations when moving unit to unit.”

—Student

## 4. Discussion

In consideration of the added clinical pressures on acute care units and teams during the COVID-19 pandemic, we developed and evaluated a float placement. In contrast to the previous literature where a float staff is deployed in response to patient surges and staff shortages, in this float model, the student was deployed across hospital units based on CI supervisor availability. This placement model was novel to both the McMaster University MSc (PT) Program and physical therapy team at St. Joseph’s Healthcare. Float placements offer the support of a clinical team and may be a strategy to increase the number of acute care placements at an institution. A multiple CI approach can decrease the overall time commitment required from individual CIs, reduce CI stress [8,9], and increase the number of clinicians willing and available to offer placement supervision [15]. In addition, the float placement offered CIs the opportunity to work with colleagues in a team supervision model, under the consistent guidance and leadership of the PCI, fostering collaboration and professional development.

This float placement fulfilled the acute care degree requirement and provided the student with exposure across various hospital units. Although we implemented this model secondary to extenuating circumstances, it could be generalizable and used in other rehabilitation disciplines in acute care. A float model could benefit multiple stakeholders, including students who require an acute care placement, CIs through a reduced time commitment for student supervision, and the facility could develop a larger pool of CIs to support placements (e.g., opportunities for new CIs or part-time employees).

For universities and facilities considering a float placement model, potential challenges exist. Given the multiple people and settings involved in this placement, planning and organization is essential. While this placement model offers the opportunity for vast skills development and experience, limited time in different clinical areas could restrict a student’s capacity to master specific clinical skills. Table 1 presents recommendations from our team for others who may consider implementing a similar model.

### Limitations

The authors recognize limitations of the float placement and of our case report. In the float placement, students have limited time to develop complex skills. Our placement occurred in an institution with a physical therapy department, which facilitated inter-therapist coordination. This placement included one student, which may limit the applicability of findings. Further study in institutions across disciplines with multiple student engagement is needed to support the experiences reported in this paper.

## 5. Conclusions

The global pandemic exacerbated the existing challenges to placement recruitment. Our float placement allowed clinicians to offer in-person supervision despite re-deployment of staff, increased caseload, organizational pressures, and complex work–life demands during the global pandemic. The float placement offers a variety of clinical experiences under the supervision of multiple CIs that may not be afforded with more traditional 1:1 (supervisor to student) placement models. Our experience provides other academic institutions and facilities with factors for consideration if they choose to develop similar placement models. Lessons learned from this successful implementation could be used to establish future models of clinical supervision in response to extenuating circumstances and increase the number of acute placements available in physical therapy and other healthcare professions with similar educational needs.

## Figures and Tables

**Table 1:** Ten tips to organize and execute the float placement.

Phase	Tip	Consideration
Organization of Placement (1–5)	1. We recommend the site plan the placement early and include a minimum of two consecutive weeks for each rotation if possible.	Attempt to include CIs that work in similar units to allow transfer of skills from one rotation to the next and consider complexities of units and patient trajectory through the site.
	2. We recommend the site provide information related to placement organization including timing of rotations and units to the university program. Ideally, this information is made available to the student in advance of the placement match.	Helps program to understand placement organization and student to self-identify required qualities to maximize success on placement and prepare for the types of clinical settings.
	3. We recommend the site designate roles for supervision including the role of the PCI.	Identify one primary clinical instructor to act as a point contact and oversee evaluations and all communication for the student, program and all supervising CIs.
	4. Following student assignment, we recommend the PCI and student identify overarching goals that can be applied to all rotations.	Identification of common learning goals across units will assist in evaluation of the clinical placement.
	5. We recommend the CIs prepare an outline for each rotation and suggest resources that will assist the student with onboarding.	Planning an outline for each rotation allows the student to prepare for placement and content that will be covered.
Execution of Placement (6–10)	6. We recommend the PCI and student review and modify overarching goals and the CI and student review the placement outline at the beginning of each rotation.	Allows revisions to be made to the overall placement goals and learning objectives within each rotation based on caseload and potential learning opportunities.
	7. We recommend the CI include one to two days of orientation at the beginning of each rotation.	Orientation to a unit and unit-specific protocols and caseload allows the student and CI to acclimate to learning environment, personalities, and associated processes.
	8. We recommend the PCI, CIs and student ensure communication and planning opportunities for frequent and timely feedback at the beginning of each placement rotation.	Assists in managing expectations and review of overarching goals. Providing and receiving timely feedback is important when placement rotations are shorter in duration than a typical placement.
	9. We recommend the PCI, CIs and student plan transition meetings between placement rotations with opportunities to provide feedback.	Allows CIs and students to review skills that have been covered/developed in each placement rotation and areas that require growth.
	10. We recommend the PCI and CI plan communications to ensure evaluations are informed by supervising CIs.	Ensures evaluations are reflective of all CI feedback.Program can support to ensure that all CIs are aware of expected benchmarks for the respective placement period.

CI = Clinical Instructor; PCI = Primary Clinical Instructor.

## Data Availability

The data presented in this paper are not publicly available due to privacy and ethical restrictions. The primary purpose of the collection and analysis of data are for program evaluation.

## Appendix A. Learning Goals

Student Goals from Learning Contract

(1) Be able to list the components of and perform a subjective assessment and understand how that subjective assessment is adapted based on unit by 2 October 2020.
(2) Be able to list the components of and perform an objective assessment and understand how that objective assessment is adapted based on unit by 2 October 2020.
(3) Able to set up patient room for mobility in all settings covered in placement with minimal to no cueing by 2 October 2020.
(4) Able to understand and identify appropriate treatment goals and plan for at least six patients by 2 October 2020.
(5) Able to identify an appropriate discharge destination and understand the discharge steps for patients across units.

## Appendix B. Reflection Questions Provided to CIs and Student

Pre-Placement

1. Given the unique placement model, did you have any expectations prior to the placement start? Please explain what these were.

Considerations in response to this question;

(6) Were there specific expectations you had of the student?
(7) Were there specific expectations you have of the overall experience?
(8) What potential barriers did you expect with this placement model?
(9) What potential opportunities did you expect with this placement model?

Placement Period

(10) Please describe the placement experience (a) week one (b) week two

Considerations in response to this question;

(11) Did your experience align with your expectations of this model?

Post Placement

(12) What are some of your general reflections on this placement model and the overall experience?

**Considerations in response to this question;**

(13) What worked well with the organization and execution of the placement (1) for the student and (2) for the Clinical Instructor
(14) What would you modify with the organization and execution of the placement to improve the overall experience (1) for the student and (2) for the Clinical Instructor?
(15) Are there any specific qualities that you think a (1) student (2) Clinical Instructor should develop/possess to assist in the delivery and execution of this placement model?
(16) Would you be involved in a placement model that is the same or similar to this in the future? Please expand on your response.
